# Supplementary material for: Combining country indicators and individual variables to predict soil-transmitted helminth infections among migrant populations: A case study from southern Italy
Source: PLoS Negl Trop Dis. 2025 Jun 13;19(6):e0012577. doi: 10.1371/journal.pntd.0012577 (PMC12208482; doi:10.1371/journal.pntd.0012577)
Supplement: S1 File — (PDF) [file pntd.0012577.s001.pdf]

Y_{ijk} represents the outcome that individual k, from country j, is infected with species i. k can take values from 1 to 3830, j can take values from 1 to 64 and i can take values 1, 2, or 3 where 1 represents *A.lumbricoides*, 2 represents hookworms and 3 represents *T. trichiura*. Y_{ijk} takes value 1 when the individual is infected, and 0 when they are not. P_{ijk} represents the probability that individual k, from country j, is infected with species i. The models we propose are as follows:

$$Y_{ijk} | U_{ijk}, V_k \sim Bernoulli\left(P_{ijk}\right)$$
$$M1: \frac{P_{ijk}}{1 - P_{ijk}} = I + M_{ijk}$$
$$M2: \frac{P_{ijk}}{1 - P_{ijk}} = I + C_{ij}$$
$$M3: \frac{P_{ijk}}{1 - P_{ijk}} = I + M_{ijk} + C_{ij}$$

I represents the intercept term in the model, which varies by species. M_{ijk} is the individual-level information, and C_{ij} are the country-level indicators. The equations for M_{ijk} and C_{ij} are as follows:

$M_{ijk} = X_{ijk}{}^T \beta_i + V_k$ where $\beta_i = \left(\beta_{1_i}, \beta_{2_i}, \beta_{3_i}\right)$ and $V_k \sim Guassian\left(0, \sigma_1\right)$.

β_{1_i} is the coefficient for age, β_{2_i} is the coefficient for the time spent in Italy, and β_{3_i} is the coefficient for sex, all these coefficients vary by species. V_k represent individual random effects.

$C_{ijk} = Z_{ij}{}^T \beta_i + U_{ij}$ where $\beta_i = \left(\beta_{1_i}, \beta_{2_i}, \beta_{3_i}, \beta_{4_i}\right)$ and $U_{ij} \sim Guassian\left(0, \sigma_2\right)$.

β_{1_i} is the coefficient for life expectation, β_{2_i} is the coefficient for GNI per Capita, β_{3_i} is the coefficient for population, and β_{4_i} is the coefficient for sanitation. All the coefficients vary by species. U_{ij} represent the country random effects.
