# Supplementary material for: Combining country indicators and individual variables to predict soil-transmitted helminth infections among migrant populations: A case study from southern Italy
Source: PLoS Negl Trop Dis. 2025 Jun 13;19(6):e0012577. doi: 10.1371/journal.pntd.0012577 (PMC12208482; doi:10.1371/journal.pntd.0012577)
Supplement: S2 File — (PDF) [file pntd.0012577.s002.pdf]

The theory of generalized U-statistic can be applied to compare two AUCs. The Mann-Whitney two-sample statistic for AUC can be written as:

$$\widehat{\theta} = \frac{1}{mn}\sum_{j=1}^{n}\sum_{i=1}^{m}\psi\left(X_i, Y_j\right)$$

where

$$\psi\left(X_i, Y_j\right) = \begin{matrix} 1 & Y<X \\ \frac{1}{2} & Y=X \\ 0 & Y>X \end{matrix}$$

m is the number of truly positive individuals and n is the number of truly negative individuals. X_i is the prediction from the model for the ith truly positive individual, and Y_j is the prediction from the model for the jth truly negative individual. This realization enables us to find the confidence interval of $\widehat{\theta}$ as follows: $\widehat{\theta} \pm 1.96 \cdot SE(\widehat{\theta})$.

To compare two AUC values, we let $\widehat{\theta}_1$ be the estimation of the AUC for model 1 and $\widehat{\theta_2}$ be the estimation of the AUC for model 2. The DeLong test uses the test statistic:

$$Z = \frac{\widehat{\theta}_1 - \widehat{\theta}_2}{\sqrt{var\left(\widehat{\theta}_1\right) + var\left(\widehat{\theta}_2\right) - 2cov\left(\widehat{\theta}_1, \widehat{\theta}_2\right)}}$$

where, due to the properties of the U-statistic, Z follows a normal distribution under the null hypothesis that $\widehat{\theta}_1 = \widehat{\theta}_2$.
