# Supplementary figures and images for: Combining country indicators and individual variables to predict soil-transmitted helminth infections among migrant populations: A case study from southern Italy
Source: PLoS Negl Trop Dis. 2025 Jun 13;19(6):e0012577. doi: 10.1371/journal.pntd.0012577 (PMC12208482; doi:10.1371/journal.pntd.0012577)

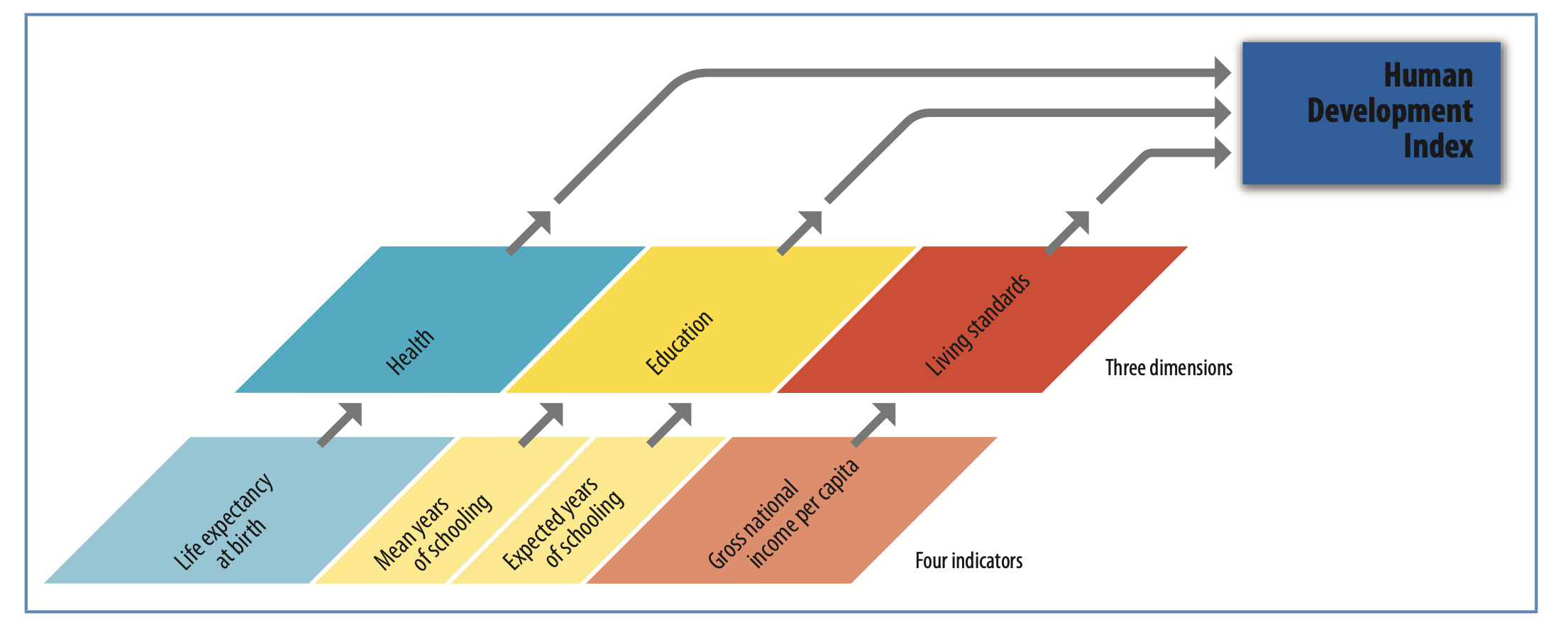

Supplement: S1 Fig — Source [19]. (TIFF) [file pntd.0012577.s003.tiff]

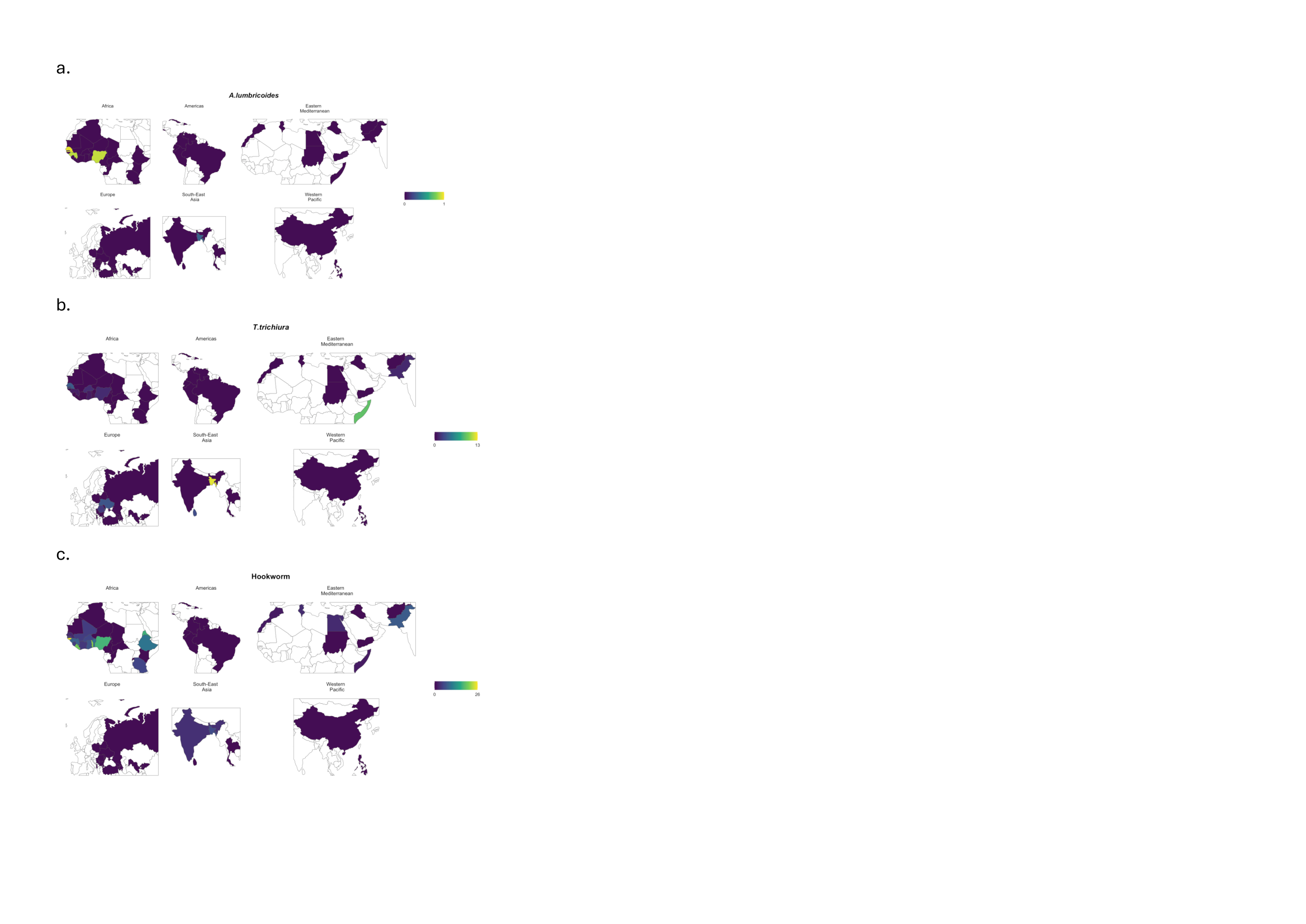

Supplement: S2 Fig — Made using Natural Earth a) A. lumbricoides, b) hookworm and c) T. trichiura. (TIFF) [file pntd.0012577.s004.tiff]

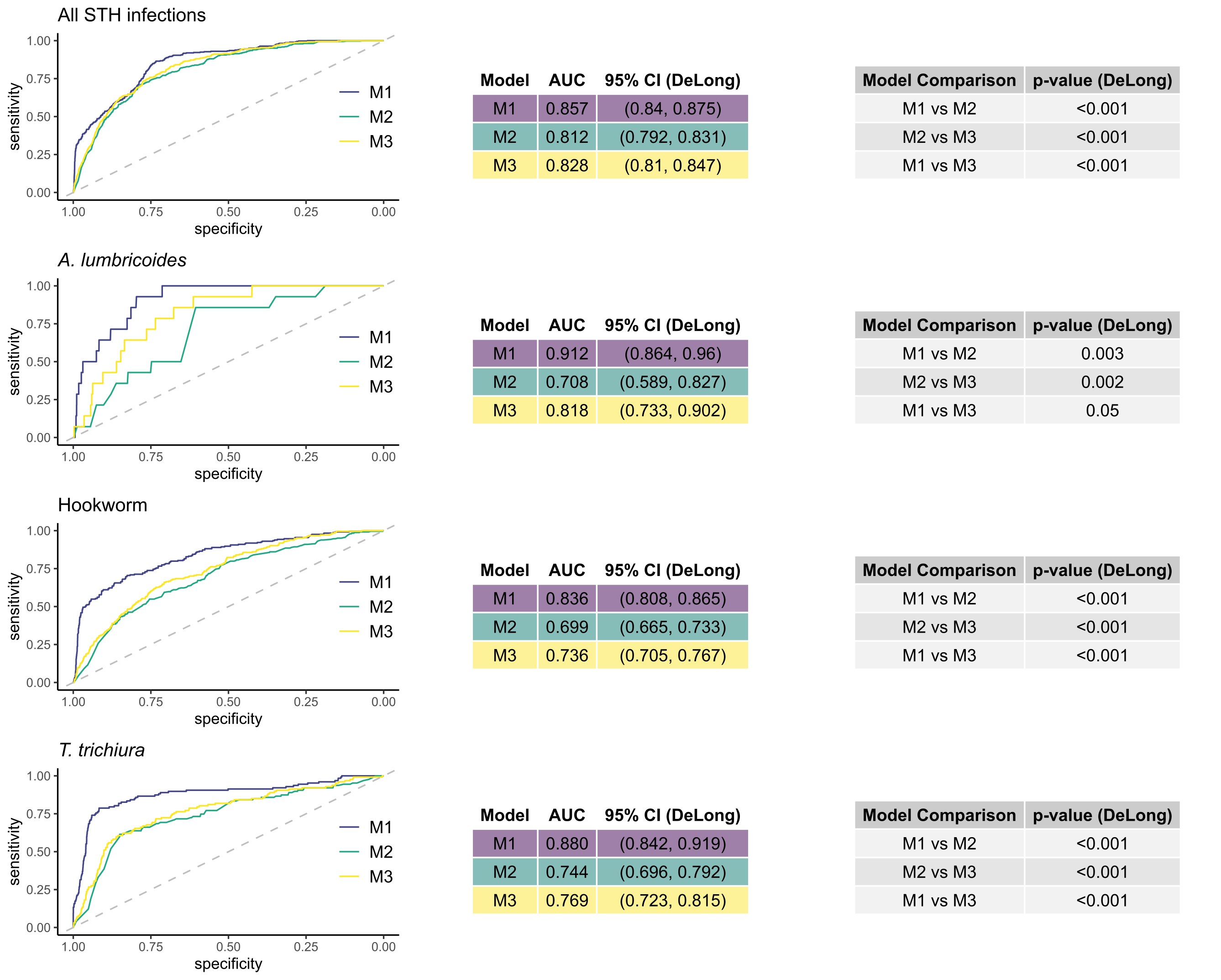

Supplement: S3 Fig — The yellow lines show the ROC for the full model, the green shows the country-only model, and the purple shows the individual-only model. AUC values and 95% CI are provided for each model in a table to the right of the plot alongside a table for each pairwise comparison of the models. (TIFF) [file pntd.0012577.s005.tiff]
