# Supplementary material for: Combining country indicators and individual variables to predict soil-transmitted helminth infections among migrant populations: A case study from southern Italy
Source: PLoS Negl Trop Dis. 2025 Jun 13;19(6):e0012577. doi: 10.1371/journal.pntd.0012577 (PMC12208482; doi:10.1371/journal.pntd.0012577)
Supplement: S1 Table — (PDF) [file pntd.0012577.s006.pdf]

Model	Random Effect	Variance
M1	Individual	0.362
M2	Country	0.201
M3	Individual	0.0188
	Country	0.227
