# Supplementary material for: Combining country indicators and individual variables to predict soil-transmitted helminth infections among migrant populations: A case study from southern Italy
Source: PLoS Negl Trop Dis. 2025 Jun 13;19(6):e0012577. doi: 10.1371/journal.pntd.0012577 (PMC12208482; doi:10.1371/journal.pntd.0012577)
Supplement: S2 Table — (PDF) [file pntd.0012577.s007.pdf]

Model	Species	Intercept Value (Odds Scale)	95% confidence interval
M1	*A.lumbricoides*	0.00237	(0.000497,0.01130)
	Hookworm	0.06120	(0.052300, 0.07150)
	T.trichiura	0.03560	(0.029400,0.04310)
M2	*A.lumbricoides*	0.00261	(0.001150, 0.00592)
	Hookworm	0.05850	(0.050300, 0.06820)
	T.trichiura	0.02270	(0.017400,0.02960)
M3	*A.lumbricoides*	0.00178	(0.000338, 0.00940)
	Hookworm	0.05570	(0.047200, 0.06570)
	T.trichiura	0.02200	(0.016700, 0.02900)
