# Supplementary material for: Combining country indicators and individual variables to predict soil-transmitted helminth infections among migrant populations: A case study from southern Italy
Source: PLoS Negl Trop Dis. 2025 Jun 13;19(6):e0012577. doi: 10.1371/journal.pntd.0012577 (PMC12208482; doi:10.1371/journal.pntd.0012577)
Supplement: S3 Table — The thresholds are based on the Euclidean distance from (0,1). (PDF) [file pntd.0012577.s008.pdf]

	M1			M2			M3		
	Threshold	Specificity	Sensitivity	Threshold	Specificity	Sensitivity	Threshold	Specificity	Sensitivity
All Infections: Existing Countries	0.04830	0.800	0.676	0.03980	0.764	0.796	0.03520	0.750	0.733
A.lumbricoides Infections: Existing Countries	0.00379	0.556	0.833	0.00278	0.651	0.830	0.00339	0.735	0.785
Hookworm Infections: Existing Countries	0.06750	0.626	0.680	0.05980	0.719	0.642	0.05840	0.718	0.692
T.trichiura Infections: Existing Countries	0.03310	0.558	0.624	0.00945	0.810	0.418	0.02440	0.545	0.507
All Infections: New Countries	0.04830	0.800	0.676	0.04160	0.745	0.813	0.04080	0.756	0.753
A.lumbricoides Infections: New Countries	0.00379	0.556	0.833	0.00316	0.640	0.839	0.00412	0.739	0.682
Hookworm Infections: New Countries	0.06750	0.626	0.680	0.06120	0.639	0.748	0.06160	0.662	0.730
T.trichiura Infections: New Countries	0.03310	0.558	0.624	0.01080	0.828	0.400	0.01360	0.715	0.421
