# Supplementary material for: Combining country indicators and individual variables to predict soil-transmitted helminth infections among migrant populations: A case study from southern Italy
Source: PLoS Negl Trop Dis. 2025 Jun 13;19(6):e0012577. doi: 10.1371/journal.pntd.0012577 (PMC12208482; doi:10.1371/journal.pntd.0012577)
Supplement: Attachment — Submitted filename: Response to Reviewers.docx [file pntd.0012577.s010.docx]

Dear Editors & Reviewers,

Thank you kindly for reviewing our paper. Please find in the documentation an updated manuscript. The tracked changes document has changes highlighted in red. We would like to address specific points you raised, noting that the line numbers are referencing the original manuscript on which the comments were made rather than the updated one

Changes have been made for clarification on the following points, as recommended:

- In the Materials and Methods, at line 101 “patients were not taking drugs or other substances”, specify how long ago and what “substances” you are referring to; at line 111, indicate that one stool sample for patient was analyzed.
- Line 172. when available?
- Line 592. it is not clear (for example because of this study given??) please modify
- Lines 89- 90. the objective is not very clear please modify also using third person
- line 92. not clear either or both

The following wording has been changed, as recommended:

- Line 541, change “were” into “was”.
- Line 545, change “the number of migrants who did not give their consent to be in the study was not recorded.” into “the migrants who did not give their consent to be in the study were not recorded.”
- Lines 555-556, change “Although the species of NTDs investigated in Martelli et al. were different from…..” into “Although the etiology of NTDs investigated in Martelli et al. was different from…….”.
- Line 107. delete since they arrive in Italy
- Line 141. yet that
- Line 145 , before respectively
- Line 174. Making
- Line 203. delete "by the" (before mean)
- Line 306. Three
- Line 373 we described
- Line 378 but had
- Line 467. . instead of ;
- Line 487 field
- line 494 [53]
- Line 551. Were
- Line 556. in that study
- Line 558. the present study
- Line 562. Suggested
- Line 585. using or utilising
- Line 589. delete another NTD
- Line 615. here we presented

The following required additional information, this has now been added:

- Line 38 – 39 is there any evidence of emergence of NTD in developed countries
- Line 54. please ad a reference.
- Line 60. please ad a reference.
- In the results section, I suggest to briefly report some more data on the prevalence of STHs in migrants coming from countries of Africa and South-East Asia indicated in yellow in Fig. 4.

The overall layout has been adjusted to take account of the following:

- Line 348 - 352. this paragraph seems repeated from the methods.
- Line 467. The first paragraph of the discussion should be the main finding related to the main objective.
- Line 574 - 580 this seems repeated from methods and results. delete of modify

There were two additional points which are not included above.

In the conclusions section of the comments, one of the reviewers has written “Authors stated this is important to minimise xenophobia and discrimination, I should suggest to use a different term such as to avoid.” This comment seems incomplete, and we are not sure what the reviewer is recommending. We would be happy to consider a change if some clarification on this point can be provided.

It was suggested that the “results could be better laid out to emphasise the predictive value of the models.”. We have carefully considered this piece of feedback, but upon reflection we believe the current layout provides a clear structure, with subheadings making it clear where we address the predictive power of the models, and how this assessment fitted into the wider process.

Thank you again for taking the time to review our paper and to provide thorough feedback, we really appreciate it.
